# A randomized, controlled trial of multi-nutrients in bipolar disorder

**DOI:** 10.1192/j.eurpsy.2026.10705

**Published:** 2026-07-31

**Authors:** L. Mehl-Madrona, B. J. Mainguy

**Affiliations:** 1 Psychiatry Residency, Northern Light Health, Bangor; 2 Family Medicine, University of New England, Portland; 3 Family Medicine Residency, Northern Light Health, Bangor; 4 Research, Coyote Institute; 5 Philosophy, University of Maine; 6 Psychotherapy, Maine Street Medicine Works, LLC; 7 Education, Coyote Institute, Orono, United States

## Abstract

**Introduction:**

An open label trial had suggested that a comprehensive micronutrient supplement, Empower Plus Advanced, in combination with Fish Oil, would be helpful in reducing symptom burden in adults diagnosed with bipolar disorder.

**Objectives:**

We aimed to determine if multi-nutrients would be an effective adjunct to pharmacotherapy in bipolar disorder.

**Methods:**

We designed a double-blinded, randomized, controlled feasibility trial. We enrolled 97 participants and randomized in a 3:2 ratio to Multi-nutrients or Placebo. We recruited patients from a family medicine residency clinic in Maine who were diagnosed in the electronic health record as having bipolar disorder. Diagnoses were confirmed via psychiatric interview or chart review (for obvious cases). The primary outcome measure was change on a composite z-score combining changes on the clinical global impressions scale (CGI), changes on the UKU Side Effects Scale, and changes in medication doses. Secondary measures included the Montgomery-Asburg Depression Scale, the Young Mania Scale (YMS), and the Brief Psychiatric Rating Scale (BPRS). Products used were Empower Plus Advanced Formula (True Hope, Inc.) and Wiley’s Alaskan Fish Oil. Placebo consisted of olive oil capsules and an inert powder with enough vitamin B complex added to change the urine color. Placebo was provided by the manufacturers of the products to mimic appearance.

**Results:**

A total of 69 patients were randomized and data was analyzed for 49 patients. The mean difference of the composite z-score for the Multi-nutrient group was 0.93 (SD; 0.35) and for the Placebo Group 0.47(SD=0.22). Independent samples t-testing gave a t-score for that difference of 2.894 (p = 0.003). In non-parametric chi-square analysis, significantly more patients in the Nutrient group improved on the CGI over the course of their participation in the study (p = 0.04; OR = 4.0; 52% responders vs. 22% in the Placebo Group). For the secondary outcome measurements, the average improvement was statistically significant between the two groups on the MADRS, the UKU, the CGI, and the BPRS. The difference in the YMS was not significant. Patients in both groups made significant improvement over time in all measures. The only adverse events occurring more among the Multi-nutrient group were nausea and loose stools (not statistically significant).

**Conclusions:**

Multi-nutrients show promise for adjunctive treatment of bipolar disorder. We observed substantial benefits for all patients of closer surveillance, medication adjustment (mostly reduction), and increased human contact. Future studies would benefit from use of a longer lead-in period during which medications can be adjusted and participants can decide if they are willing to take Multi-nutrients for an extended time. Our data suggest that primary care patients with bipolar disorder would fare better on lower medications doses and more frequent visits. Further clinical trials are warranted.

**Disclosure of Interest:**

None Declared

